# Clinical and Biochemical Predictors of Thyroid Autoantibody Levels in Hashimoto's Thyroiditis

**DOI:** 10.7759/cureus.114233

**Published:** 2026-08-09

**Authors:** Emina Bajric Custo, Danijel Gajic, Dzana Nuhanovic, Belkisa Izic, Sabina Cemalovic, Sabina Ademovic Dzambic, Dijana Hasic, Senada Selmanovic

**Affiliations:** 1 Department of Family Medicine, Health Center Lukavac, Lukavac, BIH; 2 Department of Family Medicine, Health Center Orasje, Orasje, BIH; 3 Clinic for Radiology and Nuclear Medicine, Department of Nuclear Medicine, University Clinical Center Tuzla, Tuzla, BIH; 4 Department of Cardiology, Cantonal Hospital "Dr. Irfan Ljubijankić", Bihac, BIH; 5 Department of Family Medicine, Public Health Center "Dr. Mustafa Šehović" Tuzla, Tuzla, BIH; 6 Department of Microbiology, Health Center Lukavac, Lukavac, BIH; 7 Department of Family Medicine, Health Center "Mustafa Sehovic" Tuzla, Tuzla, BIH

**Keywords:** hashimoto disease, thyroglobulin antibodies, thyroid autoantibodies, thyroid function tests, thyroid peroxidase antibodies

## Abstract

Background: Hashimoto's thyroiditis (HT) is the most common autoimmune thyroid disease and is characterized by the presence of thyroid peroxidase (anti-TPO) and thyroglobulin (anti-Tg) antibodies. However, the clinical and biochemical factors associated with these antibodies remain incompletely understood.

Objective: To evaluate the associations between anti-TPO and anti-Tg antibody levels and demographic, clinical, biochemical, and lifestyle-related factors in patients with HT.

Methods: This retrospective cross-sectional study included 100 patients with HT. Clinical, biochemical, and laboratory data were retrospectively collected from medical records. Group comparisons were performed using non-parametric tests, correlations were assessed using Spearman's rank analysis, and multivariable linear regression was used to identify independent predictors of log-transformed anti-TPO and anti-Tg levels.

Results: Anti-TPO levels differed significantly according to TSH status and were independently associated with higher TSH concentrations, longer disease duration, and lower BMI. Smoking was the only independent predictor of anti-Tg levels. No significant associations were observed between thyroid autoantibody levels and age, sex, vitamin D status, or family history of thyroid disease.

Conclusion: Anti-TPO and anti-Tg antibodies demonstrated distinct clinical associations in patients with HT. These findings support the complementary clinical value of both thyroid autoantibodies and should be confirmed in larger prospective multicenter studies.

## Introduction

Hashimoto's thyroiditis (HT) is one of the most common thyroid disorders today, and its main characteristic is the destruction of the follicular cells of the thyroid gland. HT is the predominant cause of hypothyroidism in developed regions [1]. The diagnosis is made based on the presence of a hypoechoic thyroid structure on ultrasound and elevated serum concentrations of thyroid autoantibodies, antibodies against thyroid peroxidase (anti-TPO) and antibodies against thyroglobulin (anti-Tg), but 5-10% of diagnosed cases of Hashimoto thyroiditis may be seronegative [2]. Approximately 95% of patients have detectable serum anti-thyroid peroxidase antibodies, while positive autoantibodies against thyroglobulin were recorded in about 60-80% of cases [3]. The disease affects women substantially more often than men and reflects a complex interaction between genetic susceptibility, immune dysregulation, hormonal factors, and environmental influences. HT has an estimated incidence of 0.3-1.5 cases per 1,000 individuals and is approximately 7 to 10 times more common in women than in men [4]. The clinical presentation of Hashimoto thyroiditis may involve one of three thyroid functional states: thyrotoxicosis, euthyroidism, or hypothyroidism [5].

Thyroid autoantibodies are central to the diagnosis and characterization of HT. Although these antibodies are widely used as diagnostic markers, their clinical interpretation remains complex. Autoantibody levels may vary considerably between patients and may not always correspond directly to thyroid hormone status, disease duration, or symptom burden. Recent evidence suggests that elevated thyroid antibody levels may also be associated with systemic inflammation, extrathyroidal symptoms, and impaired quality of life, indicating that autoantibody titers may reflect more than simple diagnostic positivity [6]. Despite advances in understanding its underlying immune processes, HT remains a chronic disorder with a variable clinical course. Progressive lymphocytic destruction of the thyroid gland can eventually lead to permanent hypothyroidism requiring lifelong thyroid hormone replacement therapy. However, several studies suggest that targeting modifiable factors may improve both immune markers and clinical outcomes. Such approaches include dietary modification, stress reduction, and selenium and vitamin D supplementation [7].

In addition to thyroid function parameters, several metabolic and lifestyle-related factors have been investigated in relation to autoimmune thyroid disease. Vitamin D has received particular attention because of its immunomodulatory role, including effects on T-cell regulation, cytokine production, and immune tolerance [8]. The relationship between vitamin D status and HT remains uncertain. Although numerous studies have examined this interaction in different populations, their findings have been inconsistent. Although most studies have examined the impact of vitamin D status on the prevalence of this autoimmune disease, recent research has also focused on the interaction with thyroid autoantibody levels [9]

Another factor that has been studied in the context of thyroid dysfunction is obesity, with numerous studies indicating an increased risk of hypothyroidism in this population, and serum thyroid hormone levels positively correlating with body mass index. However, studies investigating the association between obesity and autoimmune thyroid diseases are less consistent, with some studies even finding a lower prevalence of autoantibodies among obese patients [10].

Smoking has complex effects on thyroid physiology and autoimmune thyroid disease. Cigarette smoke contains compounds such as thiocyanate, which may interfere with thyroid hormone synthesis, while smoking-related immune modulation and oxidative stress may influence thyroid autoantibody production. Previous studies have reported inconsistent associations between smoking and thyroid autoimmunity, suggesting that its effects may differ according to thyroid disease subtype and specific autoantibody profile [11]. Although many studies have confirmed the diagnostic role of anti-TPO and anti-Tg antibodies, less is known about the clinical and biochemical factors associated with antibody titers in patients already diagnosed with HT. Identification of such associations may help to clarify patient heterogeneity and improve understanding of how thyroid function, metabolic status, environmental exposure, and disease duration relate to autoimmune activity. Accordingly, this study aimed to examine the relationships between anti-TPO and anti-Tg antibody levels and TSH status, smoking status, vitamin D levels, BMI, family history of thyroid disease, disease duration, and selected biochemical parameters in patients with Hashimoto thyroiditis.

## Materials and methods

Study design and setting

This retrospective observational study was conducted at the Department of Nuclear Medicine and Radiology, University Clinical Center Tuzla, Tuzla, Bosnia and Herzegovina. The study included patients diagnosed with HT who were evaluated and followed at the institution and was conducted over a three-month period, from April 1 to June 30, 2026. Clinical, anthropometric, and laboratory data were collected from available medical records and analyzed retrospectively.

Study population

The study included 100 patients diagnosed with HT. The diagnosis of HT was established based on clinical evaluation, thyroid function tests, and the presence of thyroid autoantibodies, including antibodies against thyroid peroxidase (anti-TPO) and/or antibodies against thyroglobulin (anti-Tg). For each patient, demographic, anthropometric, clinical, and biochemical variables were recorded, including age, sex, disease duration, family history of thyroid disease, body mass index (BMI), vitamin D level, smoking status (smoker/non-smoker), thyroid-stimulating hormone (TSH), free triiodothyronine (fT3), free thyroxine (fT4), anti-TPO, and anti-Tg levels.

The study included all consecutive eligible patients with HT who fulfilled the predefined inclusion criteria during the study period. Although the sample size was relatively modest, it represents all eligible patients with complete clinical and laboratory data available at our institution within the specified study period.

Patients were included in the study if they met the following criteria: diagnosis of HT; available serum anti-TPO and/or anti-Tg antibody measurements; available thyroid function parameters, including TSH, fT3, and fT4; available data on age, sex, BMI, smoking status, disease duration, and family history of thyroid disease; and age ≥18 years at the time of evaluation.

To reduce the influence of potential confounding factors, patients with conditions or treatments known to affect thyroid function or thyroid autoantibody levels were excluded. In addition, multivariable regression analyses were adjusted for age, BMI, TSH, disease duration, vitamin D level, smoking status, and family history of thyroid disease to further account for potential confounding effects.

Furthermore, patients who did not have complete medical documentation and who lacked essential laboratory data necessary for analysis were excluded from the study. Patients with a history of thyroid malignancy, previous thyroidectomy, Graves' disease, toxic nodular goiter, acute or subacute thyroiditis, pregnancy at the time of evaluation, or use of medications known to significantly affect thyroid function or immune status were also excluded.

Body weight and height were measured during clinical evaluation using standardized procedures. Body weight was measured using a calibrated scale (Seca GmbH, Hamburg, Germany). BMI was calculated by dividing body weight in kilograms by the square of height in meters and was expressed in kilograms per square meter (kg/m^2^). Patients were categorized according to BMI status as patients were classified as underweight (<18.5 kg/m^2^), normal weight (18.5-24.9 kg/m^2^), overweight (25.0-29.9 kg/m^2^), or obese (≥30.0 kg/m^2^).

Disease duration was defined as the time from diagnosis of HT to the time of data collection. Smoking status was categorized as smoker or non-smoker. Family history of thyroid disease was recorded as positive or negative. Thyroid function was assessed using serum TSH, fT3, and fT4 levels. Thyroid autoimmunity was evaluated using serum anti-TPO and anti-Tg antibody levels. Vitamin D status was assessed using serum vitamin D concentration. TSH status was categorized according to the laboratory reference range as low (<0.34 mIU/L), normal (0.35-4.94 mIU/L), or elevated TSH (≥4.95 mIU/L). Serum anti-TPO and anti-Tg antibody levels were measured using a chemiluminescent microparticle immunoassay (CMIA) on an Abbott immunoassay analyzer (Abbott Laboratories, Abbott Park, IL).

Ethical considerations

The study was approved by the Ethics Committee of the University Clinical Center Tuzla, Tuzla, Bosnia and Herzegovina (approval number: 02-09/2-110-2/26; date: 29.06.2026). The study was conducted in accordance with the principles of the Declaration of Helsinki. Owing to its retrospective design and the use of anonymized patient data, the requirement for written informed consent was waived by the Ethics Committee.

Statistical analysis

Continuous variables were presented as minimum and maximum values, median, and interquartile range (IQR), whereas categorical variables were presented as absolute frequencies and percentages. Because thyroid autoantibody levels showed a non-normal distribution, non-parametric statistical tests were used. Anti-TPO and anti-Tg levels were compared between two groups using the Mann-Whitney U test and among three or more groups using the Kruskal-Wallis test. When statistically significant differences were found across multiple groups, post-hoc pairwise comparisons were performed with Bonferroni correction.

Spearman's rank correlation coefficient was used to assess correlations between thyroid autoantibody levels and continuous clinical and biochemical variables. Multiple linear regression analysis was performed to identify independent predictors of anti-TPO and anti-Tg levels. Because anti-TPO and anti-Tg values were skewed, antibody levels were natural-log transformed before regression analysis. Regression models included age, TSH, BMI, vitamin D, disease duration, smoking status, and family history of thyroid disease as independent variables. A p-value <0.05 was considered statistically significant.

## Results

Table 1 shows the baseline characteristics of the 100 patients with HT who were included in the study. The study population was predominantly middle-aged, with a median age of 49.00 years and an age range of 21-79 years. The median disease duration was 7.00 years, indicating a heterogeneous cohort with both recently diagnosed patients and individuals with long-standing disease.

**Table 1:** Baseline characteristics of patients with Hashimoto's thyroiditis (N = 100) Abbreviations: anti-Tg: anti-thyroglobulin antibodies; anti-TPO: anti-thyroid peroxidase antibodies; BMI: body mass index; fT3: free triiodothyronine; fT4: free thyroxine; IQR: interquartile range; n: number of patients; TSH: thyroid-stimulating hormone Note: The median BMI was 26.29 kg/m^2^, placing the overall cohort within the overweight range. Thyroid function parameters showed median TSH, fT3, and fT4 values of 2.63 mIU/L, 4.42 pmol/L, and 16.15 pmol/L, respectively. Vitamin D levels were generally low to insufficient, with a median value of 21.70 nmol/L.

Variable	Minimum	Maximum	Median	IQR
Age, years	21.00	79.00	49.00	20.00
Disease duration, years	0.00	39.00	7.00	9.00
BMI, kg/m²	17.30	36.85	26.29	5.88
Vitamin D, nmol/L	6.00	66.00	21.70	11.00
TSH, mIU/L	0.02	19.79	2.63	3.73
fT3, pmol/L	2.72	6.08	4.42	0.89
fT4, pmol/L	6.96	25.01	16.15	4.30
Anti-TPO, IU/mL	3.20	1208.00	238.00	298.18
Anti-Tg, IU/mL	0.00	4000.00	72.70	288.97

The median BMI was 26.29 kg/m^2^, placing the overall cohort within the overweight range. Thyroid function parameters showed median TSH, fT3, and fT4 values of 2.63 mIU/L, 4.42, and 16.15, respectively. Vitamin D levels were generally low to insufficient, with a median value of 21.70. Thyroid autoantibody levels demonstrated substantial variability across the cohort. The median anti-TPO level was 238.00, while the median anti-Tg level was 72.70, with both markers showing wide ranges, consistent with the heterogeneity of autoimmune thyroid activity in patients with Hashimoto thyroiditis.

**Figure 1:**
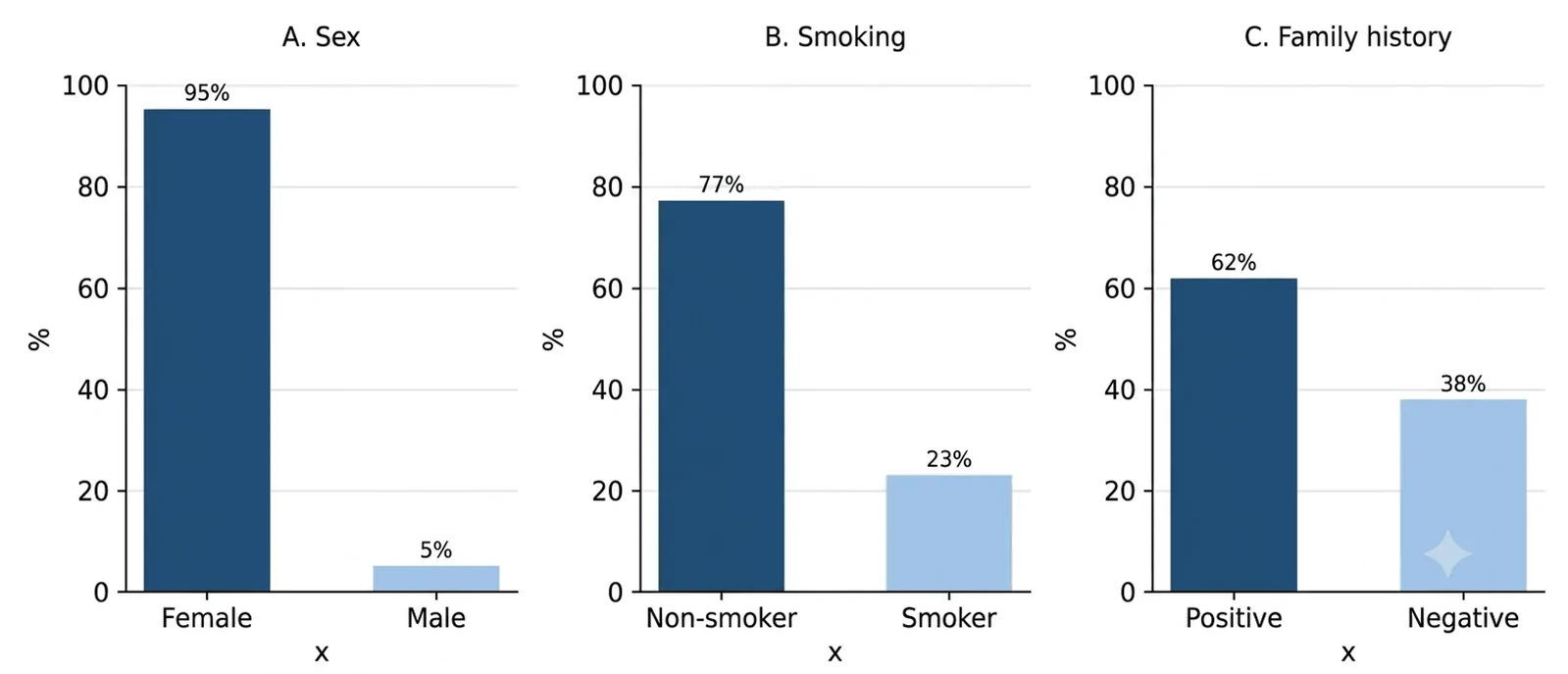
Categorical clinical characteristics of the study population

The categorical characteristics of patients with HT are presented in Figure 1. The cohort was predominantly female, with women comprising 95.0% of the study population. A positive family history of thyroid disease was reported in 62.0% of patients, while 38.0% had no reported family history. Most patients were non-smokers, accounting for 77.0% of the cohort.

Among patients with HT, anti-TPO antibody levels differed significantly across TSH categories (Kruskal-Wallis H = 7.939, df = 2, p = 0.019). The highest median anti-TPO level was observed in patients with elevated TSH. Although anti-Tg levels were numerically higher in the elevated TSH category, the difference across TSH categories was not statistically significant (Kruskal-Wallis H = 2.943, df = 2, p = 0.230), as shown in Table 2.

**Table 2:** Autoantibody levels according to TSH category in patients with Hashimoto's thyroiditis Abbreviations: anti-Tg: anti-thyroglobulin antibodies; anti-TPO: anti-thyroid peroxidase antibodies; IQR: interquartile range; n: number of patients; TSH: thyroid-stimulating hormone Note: Data are presented as min–max; median (IQR). Comparisons were performed using the Kruskal–Wallis test. Post-hoc pairwise comparisons were adjusted using Bonferroni correction. *Statistically significant at p<0.05.

Variable	TSH group	n	Minimum	Maximum	Median	IQR	Test statistic	p
Anti-TPO, IU/mL	Normal TSH (0.35-4.94 mIU/L)	70	3.20	1208.00	196.40	322.56	H = 7.939, df = 2	0.019*
	Elevated TSH (≥4.95 mIU/L)	25	23.55	1000.00	400.00	221.85		
	Low TSH (≤0.34 mIU/L)	5	66.00	272.80	160.50	171.75		
Anti-Tg, IU/mL	Normal TSH (0.35-4.94 mIU/L)	70	0.00	4000.00	67.75	238.25	H = 2.943, df = 2	0.230
	Elevated TSH (≥4.95 mIU/L)	25	1.20	4000.00	118.62	951.20		
	Low TSH (≤0.34 mIU/L)	5	0.50	202.70	44.30	183.95		

Subsequent post-hoc comparisons with Bonferroni correction revealed that anti-TPO levels were significantly higher in patients with elevated TSH compared to those with normal TSH levels (median: 400.00 vs. 196.40, adjusted p = 0.034). No other pairwise comparisons reached statistical significance after adjustment.

Thyroid autoantibody levels according to categorical clinical factors are presented in Table 3. No significant differences in anti-TPO levels were observed according to sex, family history of thyroid disease, smoking status, vitamin D category, or BMI category. No significant differences in anti-Tg levels were observed according to sex, family history, vitamin D category, or BMI category. However, anti-Tg antibody levels were significantly higher among smokers than among non-smokers, with median values of 197.80 IU/mL (IQR: 647.62) and 62.00 IU/mL (IQR: 305.15), respectively (Mann-Whitney U = 622.500, Z = -2.154, p = 0.031).

**Table 3:** Thyroid autoantibody levels according to categorical clinical factors in patients with Hashimoto's thyroiditis Abbreviations: anti-Tg: anti-thyroglobulin antibodies; anti-TPO: anti-thyroid peroxidase antibodies; BMI: body mass index; IQR: interquartile range; n: number of patients Note: Data are presented as min-max; median (IQR). Comparisons between two categories were performed using the Mann-Whitney U test, while comparisons across three or more categories were performed using the Kruskal-Wallis test. *Statistically significant at p<0.05.

Variable	Category	n	Anti-TPO median (IQR)	p	Anti-Tg median (IQR)	p
Sex	Male	5	239.50 (413.75)	0.987	44.30 (2086.05)	0.782
	Female	95	238.00 (292.70)		73.90 (315.30)	
Family history	Negative	38	216.30 (333.38)	0.524	67.95 (297.63)	0.723
	Positive	62	250.20 (288.18)		78.07 (332.24)	
Smoking status	Non-smoker	77	238.00 (319.76)	0.661	62.00 (305.15)	0.031*
	Smoker	23	238.40 (255.40)		197.80 (647.62)	
Vitamin D	Low	42	238.20 (297.29)	0.784	89.30 (224.59)	0.778
	Insufficient	36	214.85 (330.50)		67.75 (307.23)	
	Sufficient	22	261.95 (277.93)		92.81 (873.28)	
BMI	Normal weight	33	272.80 (290.70)	0.469	92.00 (283.20)	0.615
	Overweight	44	256.75 (272.05)		70.50 (273.47)	
	Obesity	22	187.05 (251.30)		76.67 (516.74)	

Spearman correlation analysis demonstrated a weak but significant positive correlation between anti-TPO and TSH concentrations (rho = 0.276, p = 0.005) and between anti-TPO and anti-Tg levels (rho = 0.228, p = 0.022). No other significant correlations were identified (Table 4).

**Table 4:** Spearman correlation analysis of anti-TPO and anti-Tg levels with clinical and biochemical parameters Abbreviations: anti-Tg: anti-thyroglobulin antibodies; anti-TPO: anti-thyroid peroxidase antibodies; BMI: body mass index; fT3: free triiodothyronine; fT4: free thyroxine; TSH: thyroid-stimulating hormone *Statistically significant at p<0.05.

Variable	anti-TPO (rho)	p-value	anti-Tg (rho)	p-value
Age	0.101	0.315	0.169	0.093
Disease duration	0.134	0.184	0.096	0.344
BMI	-0.155	0.123	0.136	0.177
Vitamin D	0.057	0.572	0.012	0.903
TSH	0.276	0.005*	0.156	0.122
fT3	-0.139	0.169	-0.030	0.765
fT4	-0.030	0.766	-0.159	0.114
anti-TPO/anti-Tg	0.228	0.022*	0.228	0.022*

Multiple linear regression analyses are presented in Table 5, evaluating independent factors associated with log-transformed anti-TPO levels (Model 1) and log-transformed anti-Tg levels (Model 2) in patients with HT. Model 1 explained 15.3% of the variability in log anti-TPO levels, with an adjusted R² of 0.089. Higher TSH levels were independently associated with higher log anti-TPO levels (β = 0.215, p = 0.032). Disease duration was also positively associated with log anti-TPO levels (β = 0.243, p = 0.020), whereas BMI showed an inverse association (β = -0.221, p = 0.034). Age, vitamin D level, smoking status, and family history of thyroid disease were not independently associated with log anti-TPO levels.

**Table 5:** Multiple linear regression models for independent determinants of log-transformed anti-TPO (Model 1) and anti-Tg (Model 2) levels in patients with Hashimoto's thyroiditis Model 1 summary: R² = 0.153; adjusted R² = 0.089. Model 2 summary: R = 0.358; R² = 0.128; adjusted R² = 0.062 Abbreviations: BMI: body mass index; CI: confidence interval; TSH: thyroid-stimulating hormone; VIF: variance inflation factor. Note: The dependent variable was the natural-log transformed anti-TPO level and anti-Tg. β represents the standardized regression coefficient. *Statistically significant at p<0.05.

Variables	Model 1					Model 2				
Predictor	B	β	95% CI	p	VIF	B	β	95% CI	p	VIF
Age	0.009	0.110	-0.008 to 0.025	0.294	1.185	0.024	0.148	-0.010 to 0.059	0.167	1.185
TSH	0.061	0.215	0.005 to 0.117	0.032*	1.060	0.099	0.167	-0.019 to 0.217	0.100	1.060
BMI	-0.055	-0.221	-0.106 to -0.004	0.034*	1.145	0.104	0.200	-0.004 to 0.212	0.058	1.145
Vitamin D	0.004	0.040	-0.015 to 0.023	0.683	1.044	0.003	0.015	-0.037 to 0.044	0.880	1.044
Disease duration	0.036	0.243	0.006 to 0.067	0.020*	1.154	-0.013	-0.040	-0.077 to 0.052	0.700	1.154
Smoking status	0.023	0.009	-0.452 to 0.498	0.924	1.026	1.176	0.229	0.170 to 2.182	0.022*	1.026
Family history	0.196	0.092	-0.245 to 0.638	0.379	1.182	0.572	0.129	-0.364 to 1.508	0.228	1.182

Model 2 explained 12.8% of the variability in log anti-Tg levels, with an adjusted R² of 0.062. Smoking status was the only statistically significant independent predictor of log anti-Tg levels (β = 0.229, p = 0.022), indicating that smokers had higher anti-Tg levels after adjustment for age, TSH, BMI, vitamin D, disease duration, and family history of thyroid disease. BMI showed a borderline positive association with log anti-Tg levels (β = 0.200, p = 0.058), whereas the remaining variables were not significantly associated with anti-Tg levels.

## Discussion

In this retrospective study involving 100 patients with HT, anti-TPO and anti-Tg antibody levels exhibited distinct associations with clinical, biochemical, and lifestyle-related factors. Anti-TPO levels were associated with TSH status, higher TSH, longer disease duration, and lower BMI, whereas anti-Tg levels were independently associated only with smoking status. These findings suggest that anti-TPO and anti-Tg may reflect partially different aspects of thyroid autoimmunity.

The demographic profile of this cohort is consistent with the known epidemiology of HT. In this study, women represented 95.0% (n = 95) of the study population, which is consistent with recent clinical abstracts reporting that HT is significantly more prevalent among women and that the female-to-male ratio can range from approximately 7:1 to 10:1 [12]. Chaker et al., in their study on hypothyroidism, state that the incidence of HT increases with age, and the largest number of cases are diagnosed between 45 and 55 years, which corresponds to the mean age of the subjects in the present study, where the median was 49 years, with a range of 21-79 years [13].

One of the principal findings of the present study was a significant relationship between anti-TPO levels and TSH status. Anti-TPO levels differed significantly across TSH categories, with the highest median values observed among patients with elevated TSH. Post-hoc analysis showed that anti-TPO levels were significantly higher among patients with elevated TSH than among those with normal TSH levels. In addition, Spearman correlation analysis revealed a weak but statistically significant positive correlation between anti-TPO and TSH, and multiple regression confirmed TSH as an independent predictor of log-transformed anti-TPO levels. The positive association between anti-TPO and TSH observed in our study is consistent with recent evidence by Vasilj-Mihaljević et al., showing a positive correlation between TPO-Ab and TSH concentrations across the euthyroid and hypothyroid ranges, as well as a negative correlation between TPO-Ab and FT4 concentrations [14].

Anti-Tg showed a different pattern. Although anti-Tg levels were numerically higher among patients with elevated TSH, the difference did not reach statistical significance, and TSH was not an independent predictor of log-transformed anti-Tg levels. This finding is clinically relevant because it suggests that anti-Tg may not parallel TSH status in the same way as anti-TPO. A significant difference was observed in the correlation of antibody levels with smoking status. In contrast to anti-TPO antibodies, which did not differ significantly between smokers and non-smokers, anti-Tg levels were statistically significantly higher in smokers. In multiple linear regression, smoking status remained the only statistically significant independent predictor of log-transformed anti-Tg levels. This result suggests that smoking may be more strongly related to anti-Tg than anti-TPO in this cohort. In contrast to this study, the PREVEND study, which compared levels of TSH, fT3, fT4, and anti-TPO antibodies, showed that higher titers of anti-TPO antibodies were identified in smokers than in non-smokers, especially in those who consumed more than 20 cigarettes per day [15].

Disease duration was independently associated with log-transformed anti-TPO levels, although this relationship was not significant in bivariate correlation analysis. This may reflect the chronic and persistent nature of anti-TPO autoimmunity in HT. Schmidt et al. found that serum TPO-Ab levels declined in most patients with HT receiving levothyroxine therapy. However, after a mean follow-up of 50 months, antibody levels became negative in only a small proportion of patients, indicating that anti-TPO positivity may persist for several years despite treatment. Therefore, the association between longer disease duration and higher anti-TPO levels in our study should be interpreted cautiously, as antibody titers may fluctuate over time and may be influenced by treatment status and disease stage [16].

In the present study, BMI showed an inverse independent association with log-transformed anti-TPO levels. This finding contrasts with the study by Wen et al., who used the weight-adjusted waist index as a marker of central obesity and reported a positive association with HT, particularly among women [17]. The discrepancy may be explained by methodological differences, as Wen et al. analyzed a large NHANES-based population using a central obesity index, whereas our study included a smaller clinical cohort of patients with established HT and used BMI, which may not adequately reflect central adiposity or metabolic dysfunction [17]. Wen et al. noted that, although previous meta-analyses suggested a positive relationship between obesity, hypothyroidism, HT, and TPO-Ab levels, some studies found no clear correlation between BMI and TPO-Ab, especially in specific female populations [18,19]. In contrast to women, the study by Zynat et al., on 2,253 subjects, determined the association of waist circumference and abdominal obesity with the levels of anti-TPO antibodies in men [10]. Therefore, our inverse association between BMI and anti-TPO should not be interpreted as evidence that higher BMI protects against thyroid autoimmunity. Rather, it may reflect the characteristics of this specific cohort, the predominance of women, possible effects of levothyroxine therapy, differences in disease stage, or unmeasured metabolic variables, such as insulin resistance, waist circumference, lipid status, and inflammatory markers.

Although vitamin D levels were generally low in our cohort, vitamin D was not significantly associated with anti-TPO or anti-Tg levels. This differs from recent meta-analytic evidence suggesting that vitamin D supplementation may reduce thyroid autoantibody levels and improve some thyroid function parameters in patients with HT. However, supplementation studies assess treatment effects, whereas our study evaluated baseline vitamin D status, which may partly explain the absence of a significant association [20]. The lack of a significant association between vitamin D and thyroid autoantibody levels may be explained by the observational design of our study, which assessed baseline vitamin D status rather than the effect of supplementation. In addition, most patients had low vitamin D levels, potentially limiting variability and reducing the ability to detect a dose-response relationship. Therefore, our findings do not directly contradict supplementation studies, but suggest that baseline vitamin D concentration alone was not a significant predictor of anti-TPO or anti-Tg levels in this cohort.

A positive family history of thyroid disease was reported in 62.0% of patients, but it was not significantly associated with anti-TPO or anti-Tg levels. This finding may be interpreted in light of the population-based study by Kim et al., which demonstrated an increased familial risk of HT among first-degree relatives. Thus, familial predisposition may be more important for disease susceptibility than for determining autoantibody titers once HT has already been established [21].

Several limitations of this study should be acknowledged. The retrospective design limits causal interpretation, and the sample size was modest, particularly in subgroup analyses, such as male patients, low TSH, and underweight BMI categories. Treatment-related variables, including levothyroxine dose and duration, were not incorporated into the analysis, although recent evidence suggests that levothyroxine dose alone may not fully explain antibody levels. Additional factors, such as iodine intake, selenium status, vitamin D supplementation, waist circumference, lipid profile, insulin resistance, inflammatory markers, thyroid ultrasound features, and smoking intensity, were also not assessed. Finally, as this was a single-center study, the findings may have limited generalizability to other populations and healthcare settings.

Despite these limitations, the study contributes to the understanding of thyroid autoantibody heterogeneity in HT. The results suggest that anti-TPO and anti-Tg antibodies have distinct patterns of association with clinical and biochemical variables. Anti-TPO was associated with TSH status, longer disease duration, and lower BMI, while anti-Tg was independently associated with smoking status. These findings support further prospective studies with larger samples and more detailed metabolic, inflammatory, treatment-related, and lifestyle data to clarify the mechanisms underlying different thyroid autoantibody profiles in HT.

## Conclusions

This study demonstrated distinct patterns of association between thyroid autoantibody levels and clinical, biochemical, and lifestyle-related factors in patients with HT. Anti-TPO levels were associated with TSH status and were independently related to higher TSH, longer disease duration, and lower BMI. In contrast, anti-Tg levels were independently associated only with smoking status, suggesting that anti-TPO and anti-Tg may reflect different aspects of thyroid autoimmunity.

Vitamin D level, family history of thyroid disease, age, fT3, and fT4 were not significantly associated with thyroid autoantibody levels in this cohort. These findings support the separate evaluation of anti-TPO and anti-Tg antibodies in patients with HT and highlight the need for further prospective studies with larger samples and a detailed assessment of treatment status, metabolic factors, and smoking exposure. The strengths of this study include the comprehensive evaluation of both anti-TPO and anti-Tg antibodies in relation to multiple clinical, biochemical, and lifestyle-related factors using multivariable analysis. However, the findings should be interpreted in light of the study limitations, including its retrospective single-center design and relatively modest sample size. Future prospective multicenter studies with larger cohorts are warranted to validate these findings.
